# The Study of Tropical Medicine

**Published:** 1909-09-04

**Authors:** 


### THE STUDY OF TROPICAL MEDICINE.

*The London School of Tropical Medicine.*—It may interest intending applicants for colonial or other posts in the tropics if one gives a short *résumé* of what the course of instruction at the London School of Tropical Medicine consists, and how long it lasts. The sessions extend over three months each, and there are three a year; the Colonial Office requires that men selected for posts in the tropics should attend one such session, and, in addition to showing regularity in attendance, should pass the examination at the end of the course. Failure to do so involves loss of the appointment. Other students may take the examination or not, as they choose, and, if successful, they are entitled to a certificate to that effect. The courses qualify, and are useful preparations, for the Diploma in Tropical Medicine and Hygiene granted by the University of Cambridge. The student is required to attend from 10 to 4 or 5 daily during the three months. The instruction is divided into three headings : (1) the practical work in the laboratory, by far the most important and useful of the three; (2) clinical instruction in the wards and the demonstration of cases; and (3) systematic lectures. Under the first heading the student is trained in hæmatology, protozoology, bacteriology, entomology, helminthology, pathology, and morbid anatomy: the tuition is eminently practical, the student preparing, staining, and mounting his own specimens, so that when he finishes his studies he has in possession a very complete set of specimens, which serve him as standards for the future. Under the second heading comes the clinical instruction in the wards: the paucity of cases is a serious drawback in this part of the teaching, though, of course, one case studied thoroughly is of much more value than many simply glanced at, but still at the same time this is a real defect, and one which it is difficult to remedy.

*The Liverpool School of Tropical Medicine.*—This school is carried on in conjunction with the University of Liverpool and the Royal Southern Hospital, for the purposes of research and clinical teaching respectively. A special ward at the hospital has been set apart for the reception and treatment of tropical diseases, and facilities for investigation and study are given in the Johnston Laboratories of the University. The fee for a full course (lasting about three months) is 10 guineas. A hall of residence is attached to the school. A vast amount of research has been accomplished at this school in addition to the ordinary tropical courses for the younger graduate, and any medical man from the tropics who wishes a helping hand in this direction cannot do better than go there.

The University of Edinburgh grants a diploma in tropical medicine and hygiene. The examinations are held in January and July. The fees for the first and any subsequent appearance are : Practical bacteriology, £1 1s.; diseases of tropical climates, £1 1s.; tropical hygiene, £1 1s.; tropical clinical medicine, £1 1s.; total, £4 4s.

